# Ehrlichia chaffeensis antibodies in white-tailed deer, Iowa, 1994 and 1996.

**DOI:** 10.3201/eid0604.000414

**Published:** 2000

**Authors:** L Mueller-Anneling, M J Gilchrist, P S Thorne

**Affiliations:** University of Iowa College of Public Health, Iowa City, Iowa 52242-5000, USA.

## Abstract

Surveillance of 2,277 white-tailed deer for antibodies against Ehrlichia chaffeensis in Iowa showed seropositivity rates of 12.5% in 1994 and 13.9% in 1996. From 1994 to 1996, the estimated number of seropositive deer increased to 54,701 (28%). The increasing deer population and expanding tick distribution may increase risk for human monocytic ehrlichiosis.


### *Ehrlichia chaffeensis* Antibodies in White-Tailed Deer, Iowa, 1994 and 1996

**Linda Mueller-Anneling,* Mary J. Gilchrist,† and Peter S. Thorne***
*University of Iowa College of Public Health, Iowa City, Iowa, USA; and
†University of Iowa Hygienic Laboratory, Iowa City, Iowa, USA

Surveillance of 2,277 white-tailed deer for antibodies against *Ehrlichia chaffeensis* in Iowa showed seropositivity rates of 12.5% in 1994 and 13.9% in 1996. From 1994 to 1996, the estimated number of seropositive deer increased to 54,701 (28%). The increasing deer population and expanding tick distribution may increase risk for human monocytic ehrlichiosis.

Human monocytic ehrlichiosis (HME) is a newly recognized disease caused by *Ehrlichia chaffeensis*, a rickettsialike, gram-negative, pleomorphic bacterium (1). HME causes clinical illness, from mild and flulike to life threatening. A prolonged incubation period and ambiguous symptoms complicate the diagnosis. Early recognition and treatment of the disease are key to preventing death. Identifying the ecologic niches for the etiologic agent and vector is important for characterizing ehrlichiosis and the occupational and environmental risk for illness. The southern counties of Iowa border the region of endemic HME, and an important reservoir host, the white-tailed deer (*Odocoileus virginianus*), is common in Iowa, as is the vector, the Lone Star tick (*Amblyomma americanum)* (2,3).

Although humans may be hosts for nymph and adult ticks, they are not the preferred mammal for the tick and therefore are accidental dead-end hosts of HME (4). Person-to-person transmission is not known to occur. Most likely to be infected with *Ehrlichia* are populations with an increased risk for tick exposure, including hunters, farmers, hikers, campers, foresters, and park rangers. To define the prevalence and geographic distribution of *E. chaffeensis* in Iowa, we tested blood collected from white-tailed deer from every county in the state in 1994 and 1996. White-tailed deer were used as sentinel animals for HME because 1) nonspecific symptoms complicate the diagnosis of mild and subclinical cases in humans; 2) deer are primary hosts for *A. americanum*; 3) the limited home range of deer (4 $km^2$ [5]) allows accurate determination of geographic distribution; and 4) hunters in Iowa are willing to provide deer blood samples. In addition, tick collection, speciation, and analysis by polymerase chain reaction would be prohibitive for evaluation of *E. chaffeensis* in all 99 counties in Iowa. Testing ticks would likely require analysis of huge numbers of specimens to detect significant numbers of positives. However, each deer during its lifetime may be bitten by thousands of ticks, and any one of the bites may lead to infection and seroconversion.

### The Study

Randomly selected licensed deer hunters (2,500 in the 1994 and 2,000 in the 1996 hunting seasons) were sent packets containing an explanation of the study, two Nobuto blood filter strips (Toyo Roshi Kaisha, Ltd., Tokyo, Japan), and a postage-paid return envelope. Hunters were instructed to collect a deer blood sample on the Nobuto strip, allow the sample to dry, place it in a resealable bag, and mail it, along with a map showing the county where the deer was killed. The dried whole blood samples (0.1 mL) were eluted from the Nobuto strips by soaking in phosphate-buffered saline (PBS), pH 7.4, for 3 hours at 4°C. The eluted samples were stored at -70°C until tested. Twelve-well, Teflon-coated, glass microscope slides were coated with *E. chaffeensis*-infected DH82 canine malignant

Address for correspondence: Peter S. Thorne, University of Iowa, College of Public Health, Department of Occupational and Environmental Health, 100 Oakdale Campus, 176 IREH, Iowa City, IA 52242-5000, USA; fax: 319-335-4006; e-mail: peter-thorne@uiowa.edu.

macrophages. All blood specimens were analyzed by immunofluorescent antibody (IFA) assay at a dilution of 1:64 in PBS (6). Specimens with at least 1+ fluorescence were considered positive.

To calculate the statistical significance of the difference in statewide and regional prevalence rates for 1994 and 1996, chi-square tests of homogeneity of binomial proportions were used. A chi-square test for trend in binomial proportions was used to determine significance of the north-to-south trend. All tests were performed by using EpiInfo 6.04b, and an alpha <0.05 was considered significant.

Of the 4,500 blood-sampling packets mailed to hunters, 2,881 were returned, of which 2,877 were usable (64% overall: 71% in 1994 and 55% in 1996) (Table 1). All 99 counties of Iowa were represented in the 1994 samples (mean 17.9 specimens per county [2 to 82]). The 1996 samples were collected in 97 of the 99 counties (mean 11.2 specimens per county [0 to 82]). To facilitate analysis of geographic trends in seroprevalence, we divided the Iowa map horizontally into three rows of counties (Figure).

The deer population has been increasing in Iowa (Table 1). To establish estimates of statewide prevalence, population estimates of deer per county were obtained from the Iowa Department of Natural Resources (DNR) (W.J. Suchy, pers. comm.). From 1994 to 1996 the deer population in Iowa increased by 14.0%, or more than 45,000 deer. The deer population was distributed with 23.3% and 27.0% in the north in 1994 and 1996, respectively; 43.5% and 40.3% were located in the southern region in 1994 and 1996, respectively. Although the population density was higher in the south, most increases in deer population occurred in the north. This increase accounts for the difference in the regional distribution of blood samples across years (Table 1). The estimated proportions of deer in Iowa included in this study (Table 2) are 0.55% sampled in 1994 and 0.30% in 1996. The ratio of the 1994 proportion to the 1996 shows no difference in the regional distribution of population-based sampling across years (North, 1.84; Central, 1.84; South, 1.82). The statewide seroprevalence rate was 12.5% in 1994 but increased to 13.9% in 1996 (Table 2). The maps demonstrate that seropositivity followed a north-to-south gradient, with the highest prevalence rates in the southernmost counties (Figure). Six

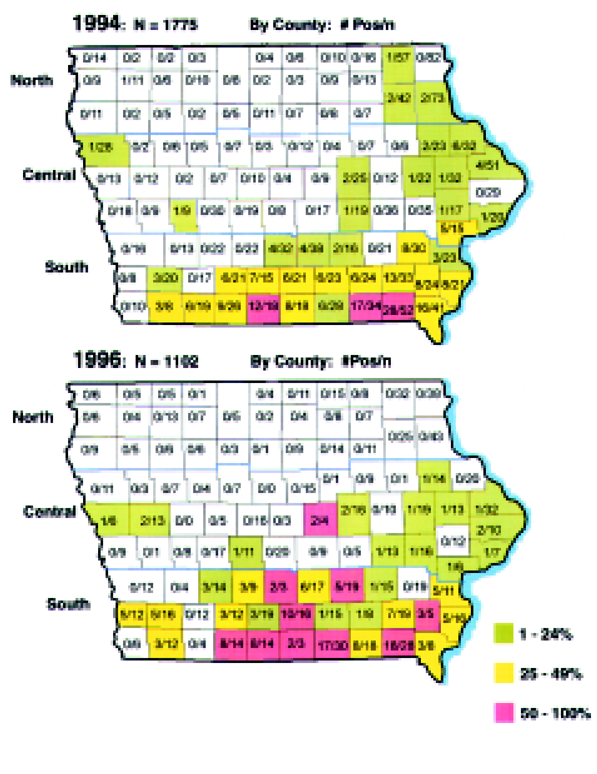

Figure. Iowa maps showing *Ehrlichia chaffeensis* seropositive specimens by county. The total number of positive specimens was 102 of 1,775 in 1994 (top map) and 91 of 1,102 in 1996 (bottom map). Within each of the 99 counties is listed the number of positive specimens over the total submitted for the county. Colors indicate the percentage of positive specimens as listed in the key.

Table 1. Geographic distribution of deer blood specimens collected and estimated deer population, Iowa, 1994 and 1996

| | 1994 | | | 1996 | | | Pop. |
|---|---|---|---|---|---|---|---|
| Region | Est. pop. | No. sampled | % sampled | Est. pop. | No. sampled | % sampled | increase % |
| North | 75,252 | 444 | 25.0 | 99,531 | 318 | 28.9 | 32.3 |
| Central | 107,344 | 617 | 34.8 | 120,134 | 374 | 33.9 | 11.9 |
| South | 140,470 | 714 | 40.2 | 148,569 | 410 | 37.2 | 5.8 |
| Iowa | 323,067 | 1,775 | 100.0 | 368,234 | 1,102 | 100.0 | 14.0 |

Table 2. Deer seropositivity for antibodies to *Ehrlichia chaffeensis*, Iowa, 1994 and 1996.

| Year | Result | North | Central | South | Statewide |
|---|---|---|---|---|---|
| 1994 | No. of samples | 444 | 617 | 714 | 1,775 |
| | % of population sampled | 0.59 | 0.57 | 0.51 | 0.55 |
| | No. positive | 6 | 26 | 189 | 221 |
| | % positive | 1.35 | 4.21 | 26.5 | 12.5 |
| | Est. no. of positive deer | 1,017 | 4,523 | 37,183 | 42,724 |
| | Odds ratio | 1.0 | 3.2 | 26.3[a] | |
| 1996 | No. of samples | 318 | 374 | 410 | 1,102 |
| | % of population sampled | 0.32 | 0.31 | 0.28 | 0.30 |
| | No. positive | 0[b] | 18 | 135 | 153 |
| | % positive | 0 | 4.81 | 32.9 | 13.9 |
| | Est. no. of positive deer | 0 | 5,782 | 48,919 | 54,701 |
| | Odds ratio | 1.0 | 16.1 | 156.1[c] | |

[a]Chi square for trend = 183, p<0.00001
[b]One positive case was assumed for this cell to allow calculation of the odds ratio
[c]Chi square for trend = 173, p<0.00001

seropositive deer in the 1994 survey and none in 1996 were found in the northern counties. Five of the six positive specimens in the north were near the Mississippi River, the eastern border of Iowa. In the central region, only one county yielded >24% positive specimens, on the basis of four samples. These findings contrast with the results from the southern region, where in 1996 seropositive deer were found in 25 of the 31 counties and 20 counties had >25% positive samples.

Seroprevalence data, odds ratios and estimated numbers of seropositive deer were calculated to determine the strength of the trend of increasing seroprevalence with location (Table 2). In both survey years, a highly significant trend was observed (1994: chi square = 183, p <0.00001; and 1996: chi square = 173, p <0.00001). Analysis of seroprevalence data in each region and for the whole state across survey years demonstrated a significant increase only in the south (chi square = 5.3, p = 0.021) (Table 2). These figures indicate a 28.0% increase in statewide seroprevalence from 1994 to 1996, attributable mostly to increases in the southern region.

### Conclusions

When prevalence rates were assessed for three regions of Iowa, a gradient of increasing seroprevalence was shown from north to south. This information can help direct occupational and public health efforts to regions of greatest risk for HME. In the southern region, one-fourth to one-third of deer were seropositive for antibodies against *E. chaffeensis*. Our data show that potential for HME is present in southern Iowa because of proximity to a disease-endemic area and the presence of the vector tick. This heavily wooded area provides ideal conditions for deer and ticks to come into contact. *E. chaffeensis*-seropositive deer were also found in the Mississippi River valley on the eastern border and near several interior rivers. These uninterrupted wet, wooded regions facilitate the spread of *E. chaffeensis* northward and westward in Iowa. The north-central area, which is relatively flat, has an abundance of deer but fewer forested areas, and the Lone Star tick is uncommon. Therefore, deer in this region are unlikely to have as much contact with the Lone Star tick as deer in southern Iowa or along wooded river valleys. Our data support the conjecture that the north-central area should be a region of sustained low prevalence of HME.

This study has several limitations. First, information on the deer population by county may have the same selection bias as our study, since much of the data are drawn from numbers of deer killed by hunters. According to Iowa DNR wildlife biologists, the actual deer population may differ from estimates by as much as 10%. Second, many counties had a limited number of specimens submitted. However, deer hunters tend to hunt where deer are most plentiful. Third, IFA has high sensitivity but unknown specificity; therefore, cross-reactions with nonpathogenic *Ehrlichia* species could occur. However, cross-reactions to species such as *E. canis* and *E. ewingii* were unlikely, and no reports indicate that deer become infected with these organisms. As an emerging pathogen, *E. chaffeensis* has only recently been studied extensively. Although human cases of disease have occurred in Illinois, Missouri, and other states south of Iowa, disease transmission in Iowa has not been recognized. Most physicians are familiar with Lyme disease and Rocky Mountain spotted fever, but few are aware of ehrlichiosis and may not include it in differential diagnoses.

### Acknowledgments

The authors thank James S. Gill, Amy Bernhard, and Iowa hunters for their assistance; Jacqueline E. Dawson for supplying the HME antigen; and the Iowa Department of Natural Resources for access to lists of licensed deer hunters.

Ms. Mueller-Anneling is a doctoral student in Dr. Thorne's laboratory in the University of Iowa College of Public Health. Dr. Thorne is Professor of Toxicology and Environmental Engineering at the University of Iowa; his research interests include bioaerosol toxicology and animal inhalation models for pulmonary diseases.

### References

1. Dumler JS, Bakken JS. Human ehrlichiosis: newly recognized infections transmitted by ticks. Annual Rev Med 1998;49:201-13.
2. Lockhart JM, Davidson WR, Stallknecht DE, Dawson JE. Site-specific geographic association between *Amblyomma americanum* (Acari: Ixodidae) infestations and *Ehrlichia chaffeensis* reactive (Rickettsiales: *Ehrlichieae)* antibodies in white-tailed deer. J Med Entomol 1996;33:153-8.
3. Ewing SA, Dawson JE, Kocan AA, Barker RW, Warner CK, Panciera RJ, et al. Experimental transmission of *Ehrlichia chaffeensis* (Rickettsiales: *Ehrlichieae)* among white-tailed deer by *Amblyomma americanum* (Acari: Ixodidae). J Med Entomol 1995;32:368-74.
4. Fishbein DB, Dawson JE, Robinson LE. Human ehrlichiosis in the United States, 1985 to 1990. Ann Intern Med 1994;120:736-43.
5. Hiller ILO. Habitat and food in the white-tailed deer. 1st ed. College Station (TX): Texas A&M University Press; 1997. p 17-8.
6. Dawson JE, Fishbein DB, Eng TR, Redus MA, Greene NR. Diagnosis of human ehrlichiosis with the indirect fluorescent antibody test: kinetics and specificity. J Infect Dis 1990;162:91-5.
